# The Burden of Colorectal Cancer Treatment on Quality of Life: A Paired Longitudinal Analysis of Medicare Advantage Enrollees

**DOI:** 10.1002/jso.28161

**Published:** 2025-06-02

**Authors:** Emna Bakillah, J. Walker Rosenthal, Solomiya Syvyk, Chris Wirtalla, James Sharpe, Raina M. Merchant, Shivan J. Mehta, Carmen E. Guerra, Rachel R. Kelz

**Affiliations:** 1 Department of Surgery, Perelman School of Medicine of the University of Pennsylvania, Philadelphia, Pennsylvania, USA; 2 Department of Surgery, Center for Surgery and Health Economics, Philadelphia, Pennsylvania, USA; 3 Leonard Davis Institute of Health Economics, University of Pennsylvania, Philadelphia, Pennsylvania, USA; 4 Department of Emergency Medicine, Perelman School of Medicine of the University of Pennsylvania, Philadelphia, Pennsylvania, USA; 5 Division of Gastroenterology, Perelman School of Medicine of the University of Pennsylvania, Philadelphia, Pennsylvania, USA; 6 Abramson Cancer Center, University of Pennsylvania, Philadelphia, Pennsylvania, USA; 7 Department of Medicine, Perelman School of Medicine of the University of Pennsylvania, Philadelphia, Pennsylvania, USA

**Keywords:** colorectal cancer treatment, health-related quality of life, Medicare advantage, outcomes

## Abstract

**Background and Objectives:**

Treatment of colorectal cancer (CRC) can have prolonged effects on health-related quality of life (HRQOL). Using the Medicare Health Outcomes Survey (MHOS), this study examines HRQOL outcomes among those undergoing CRC treatment and those who completed CRC treatment.

**Methods:**

We performed a paired longitudinal retrospective cohort study of Medicare Advantage enrollees ≥ 65 years of age who completed the baseline and follow-up MHOS from 2016 to 2020 and answered survey questions regarding current CRC treatment. Outcomes included Physical Component Summary (PCS) scores and Mental Component Summary (MCS) scores. Multivariable logistic regression analyses were used.

**Results:**

574 Respondents met the inclusion criteria. Those currently undergoing treatment for CRC had significantly lower PCS scores (*β* coefficient −3.08 points, *p* < 0.001) and significantly lower MCS scores (*β* coefficient −1.40 points, *p* = 0.008) at follow-up compared to when they were not undergoing CRC treatment at baseline. Respondents who completed CRC treatment had PCS and MCS scores that remained similar over time (*β* coefficient 0.54 points, *p* = 0.466 and 0.07 points, *p* = 0.924, respectively).

**Discussion:**

Treatment of CRC negatively influences HRQOL. These findings emphasize the importance of informing patients of the long-term effects of CRC treatment and support the implementation of interventions aimed at providing sustained recovery throughout the survivorship continuum.

## 1 | Introduction

Colorectal cancer (CRC) is the third most common cancer worldwide, accounting for 10% of all cancer cases [1]. CRC most commonly affects people over the age of 50 years [2]. Treatment is multimodal and may include surgical resection, radiotherapy, chemotherapy, and immunotherapy [3]. Due to advancements in screening and treatment [4], the 5-year relative survival rate for CRC has increased from 50% in the mid-1970s to 65% during 2012–2018 [5]. Higher survival rates combined with an aging population have resulted in more than 1.4 million CRC survivors in the United States as of January 2022 [6].

Survivorship within the context of cancer begins at the time of diagnosis and continues throughout life posttreatment [7]. Treatment of CRC can induce long-term effects on health-related quality of life (HRQOL) [8] that may persist for months or years and can be both physical and psychosocial in nature [9]. Sequelae of CRC treatment that can affect quality of life well after treatment include fatigue, physical limitations, depression, and negative body image [10].

To capture patient-reported HRQOL outcomes, the Medicare Health Outcomes Survey (MHOS) was introduced as a tool for use in Medicare managed care [11]. The purpose of the survey is to gather valid and reliable data on the health status of Medicare beneficiaries enrolled in Medicare Advantage to monitor plan performance and improve health outcomes [11]. The survey includes HRQOL outcome measurements related to both physical and mental health.

Using the MHOS, we aim to examine HRQOL outcomes among individuals undergoing CRC treatment and among individuals who have completed treatment of CRC. We hypothesize that individuals undergoing CRC treatment at follow-up will have worse HRQOL outcomes compared to their baseline, and that individuals will have improved HRQOL outcomes after the completion of treatment.

## 2 | Materials and Methods

### 2.1 | Data Source and Population

This is a paired longitudinal retrospective cohort study of adults age ≥ 65 years of age who completed the baseline Medicare Health Outcomes Surveys (MHOS) [12] from 2016 to 2020. The MHOS is a 67-item questionnaire administered annually to a random sample of Medicare Advantage enrollees. Enrollees receive a baseline survey and an identical follow-up survey 2 years later. The survey is available in English, Spanish, Chinese, and Russian. Participants complete the survey via a mailed questionnaire form or a telephone-administered interview. Questions vary in form and include scale questions, dichotomous/categorical questions, and open-ended questions.

Individuals who answered baseline and follow-up MHOS survey questions regarding current CRC treatment were included. Respondents who reported current treatment for any other type of cancer, including lung, breast, prostate, and other cancers (other than skin cancer) or who had incomplete surveys (< 80% complete) were excluded. The first cohort of respondents examined (those undergoing CRC treatment) answered “No” to current CRC treatment in the baseline survey and “Yes” to current CRC treatment in the follow-up survey. The second cohort of respondents (those who completed CRC treatment) answered “Yes” to current CRC treatment in the baseline survey and “No” to current CRC treatment in the follow-up survey.

### 2.2 | Covariates

Age, plan information, and institutional or hospice status were extracted directly from the data set. All other covariates were self-reported by the respondent by way of survey completion. Covariates related to baseline socioeconomic demographics included: age, sex, race, ethnicity, primary spoken language, survey language, proxy completion of survey, marital status, education level, household income, special needs plan, Medicaid status, and Centers for Medicaid and Medicare Services (CMS) plan region. Covariates related to baseline health status abstracted from the database included: body mass index (BMI), current smoking status, and baseline comorbidities. Baseline comorbidities included hypertension, angina pectoris or coronary artery disease, congestive heart failure, myocardial infarction or heart attack, other heart conditions, stroke, emphysema, asthma or chronic obstructive pulmonary disease, inflammatory bowel disease, end-stage renal disease, arthritis, osteoporosis, sciatica, and diabetes.

### 2.3 | Outcomes

Embedded in the MHOS is the Veterans RAND 12-item Health Survey (VR-12), which measures HRQOL. The 12 items in the VR-12 questionnaire correspond to eight principal physical and mental health domains, including general health perceptions, physical functioning, role limitations due to physical and emotional problems, bodily pain, energy-fatigue, social functioning, and mental health. Response scales are based on either 5- or 6-point ordinal choices ranging from “None of the time” to “All of the time”, or 3-point ordinal scales ranging from “Yes, limited a lot” to “No, not limited at all”; an overall health question has a 5-point ordinal response scale that ranges from Excellent” to “Poor.” The 12 items are summarized into two scores, a Physical Component Summary (PCS) score and a Mental Component Summary (MCS) score [13]. PCS and MCS scores range from 0 to 100, with 50 representing the United States population mean with a standard deviation of 10; higher scores indicate better health. A change of two points in either direction is considered clinically meaningful [14, 15]. The distribution that each health domain contributes to PCS and MCS scores varies. However, all domains contribute to some extent to the scoring of both summary measures (Supporting Information S1: Table 1).

### 2.4 | Statistical Approach

Descriptive statistics, baseline and follow-up survey scores, and comparison of outcomes were calculated within each individual cohort. Multivariable logistic regression analyses clustered by respondent identifiers were used to examine outcomes with adjustment for potential confounders. A method suggested by McCullagh for paired comparisons of ordinal categorical data was used to test differences in movement across individual questionnaire item responses that contribute to PCS and MCS scores [16]. This yielded the odds of an inferior category response on the follow-up survey compared to the baseline survey for the cohort undergoing CRC treatment, and the odds of a superior category response on the follow-up survey compared to the baseline survey for the cohort who completed CRC treatment. The study protocol was reviewed and approved by the Institutional Review Board (IRB) at the University of Pennsylvania. The study was performed in accordance with guidelines set forth by the Strengthening the Reporting of Observational Studies in Epidemiology (STROBE) initiative [17]. All statistical tests were considered significant at a two-sided level of significance of 0.05 (α ≤ 0.05), and analyses were completed in Stata 17.0 (StataCorp LLC, College Station, TX, USA), SAS 9.4 (SAS Institute, Cary, NC, USA), and R 4.2.2 (R Foundation for Statistical Computing, Vienna, Austria).

## 3 | Results

### 3.1 | Descriptive Statistics

**FIGURE 1 |:**
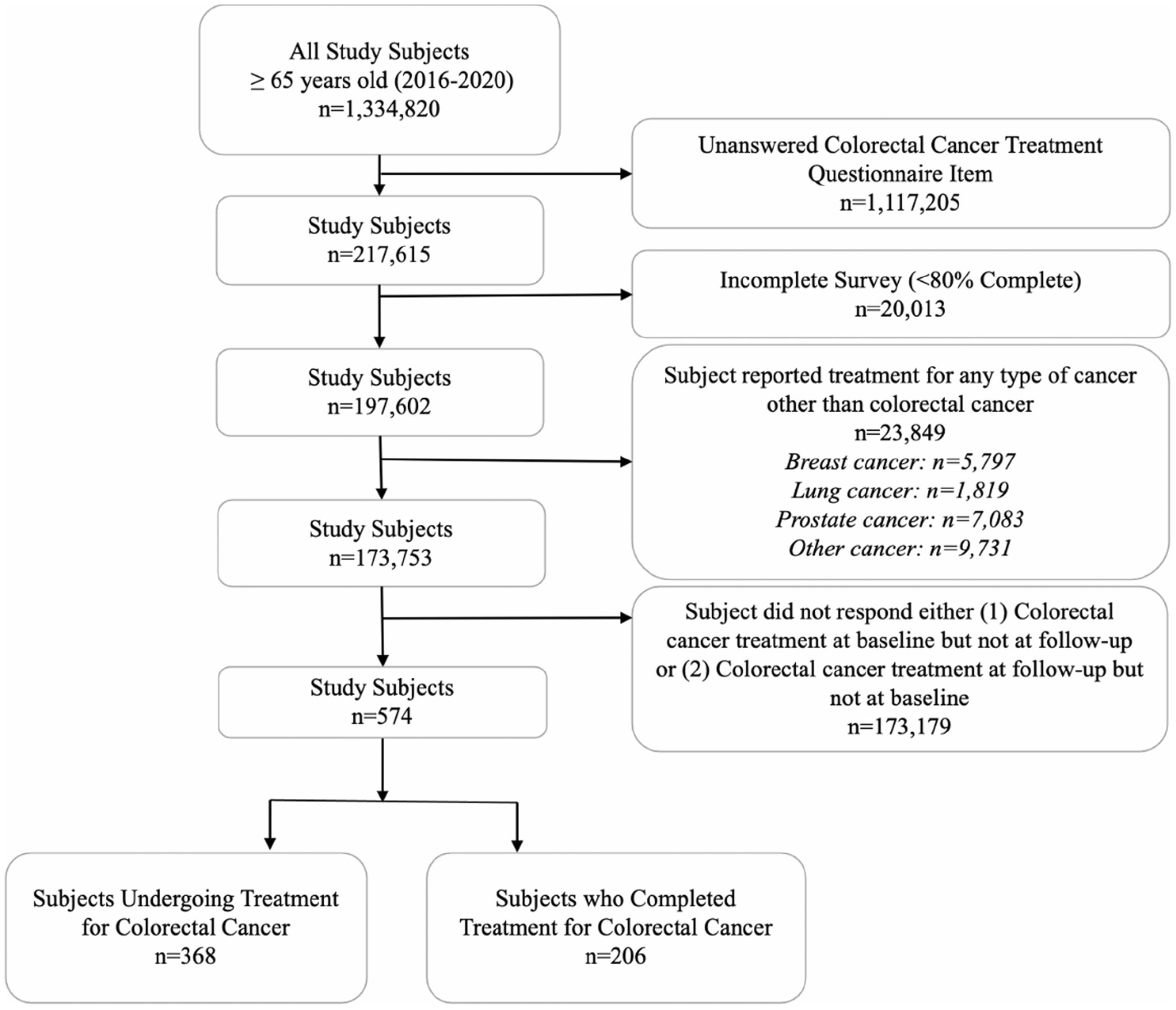
Consort diagram of subject selection.

The study population included 574 individuals (Figure 1). After exclusion criteria were applied, the rate of missing was low as data completeness was robust.

### 3.2 | Cohort Undergoing CRC Treatment

Respondents answered “No” to current CRC treatment on the baseline survey and “Yes” to current CRC treatment on the follow-up survey. This cohort included 368 adults. On average, these individuals were 74.7 years old, and 48.9% of them were female. A majority were White (78.5%) and non-Hispanic (76.4%). The cohort included 37 Black respondents (10.1%), and 23.6% of respondents were Hispanic. A majority spoke English as their primary language (82.9%) and completed the survey in English (92.9%). Thirty-five respondents spoke Spanish as their primary language (9.5%). Of the total cohort, a majority completed the survey themselves (85.1%), while 9.0% had a proxy complete the survey for them. A majority of the cohort did not have Medicaid dual eligibility status (70.7%), and there was wide variation in household income status (Table 1). With regard to baseline health conditions, a majority of respondents were overweight (30.4%) or obese (35.3%) and had a diagnosis of hypertension (66.8%) at the time of baseline survey completion (Table 2).

### 3.3 | Cohort Who Completed CRC Treatment

Respondents answered “Yes” to current CRC treatment on the baseline survey and “No” to current CRC treatment on the follow-up survey. This cohort included 206 adults. On average, these individuals were 74.6 years old, and 48.5% of them were female. A majority were White (68%) and non-Hispanic (59.7%). The cohort included 30 Black respondents (14.6%), and 40.3% were Hispanic. A majority spoke English as their primary language (70.9%) and completed the survey in English (91.7%). Thirty-eight respondents spoke Spanish primarily (18.4%). Of the total cohort, a majority completed the survey themselves (74.3%), while 14.6% had a proxy complete the survey for them. A majority of the cohort did not have Medicaid dual eligibility status (61.7%), and a majority had household incomes less than $30 000 per year (56.7%) (Table 1). With regard to baseline health conditions, a majority of respondents were overweight (28.6%) or obese (37.4%) and had a diagnosis of hypertension (73.8%) and arthritis of the hip or knee (51.9%) at the time of baseline survey completion (Table 2).

### 3.4 | Unadjusted Outcomes

The cohort undergoing CRC treatment had lower unadjusted PCS and MCS scores on follow-up survey compared to baseline. These respondents had a mean PCS score of 39.27 at baseline and a mean PCS score of 36.18 at follow-up. Furthermore, they had a mean MCS score of 52.87 at baseline and a mean MCS score of 51.47 at follow-up (Table 3).

The cohort who completed CRC treatment also had lower unadjusted PCS and MCS scores on follow-up survey compared to baseline survey. These respondents had a mean PCS score of 38.53 at baseline and a mean PCS score of 36.74 at follow-up. Furthermore, they had a mean MCS score of 52.21 at baseline and a mean MCS score of 51.34 at follow-up (Table 3).

### 3.5 | Adjusted Outcomes

After adjusting for potential confounders, respondents undergoing CRC treatment had statistically significantly lower PCS scores at follow-up compared to baseline (*β* coefficient −3.08 points, *p* < 0.001). Similarly, they had statistically significant lower MCS scores at follow-up compared to baseline (*β* coefficient −1.40 points, *p* = 0.008) (Table 4).

Respondents who completed CRC treatment did not have statistically significant differences in PCS or MCS scores at follow- up compared to baseline (*β* coefficient 0.54 points, *p* = 0.466 and *β* coefficient 0.07 points, *p* = 0.924, respectively) (Table 4).

### 3.6 | Odds of Change in Response to Individual Questionnaire Item

**FIGURE 2 |:**
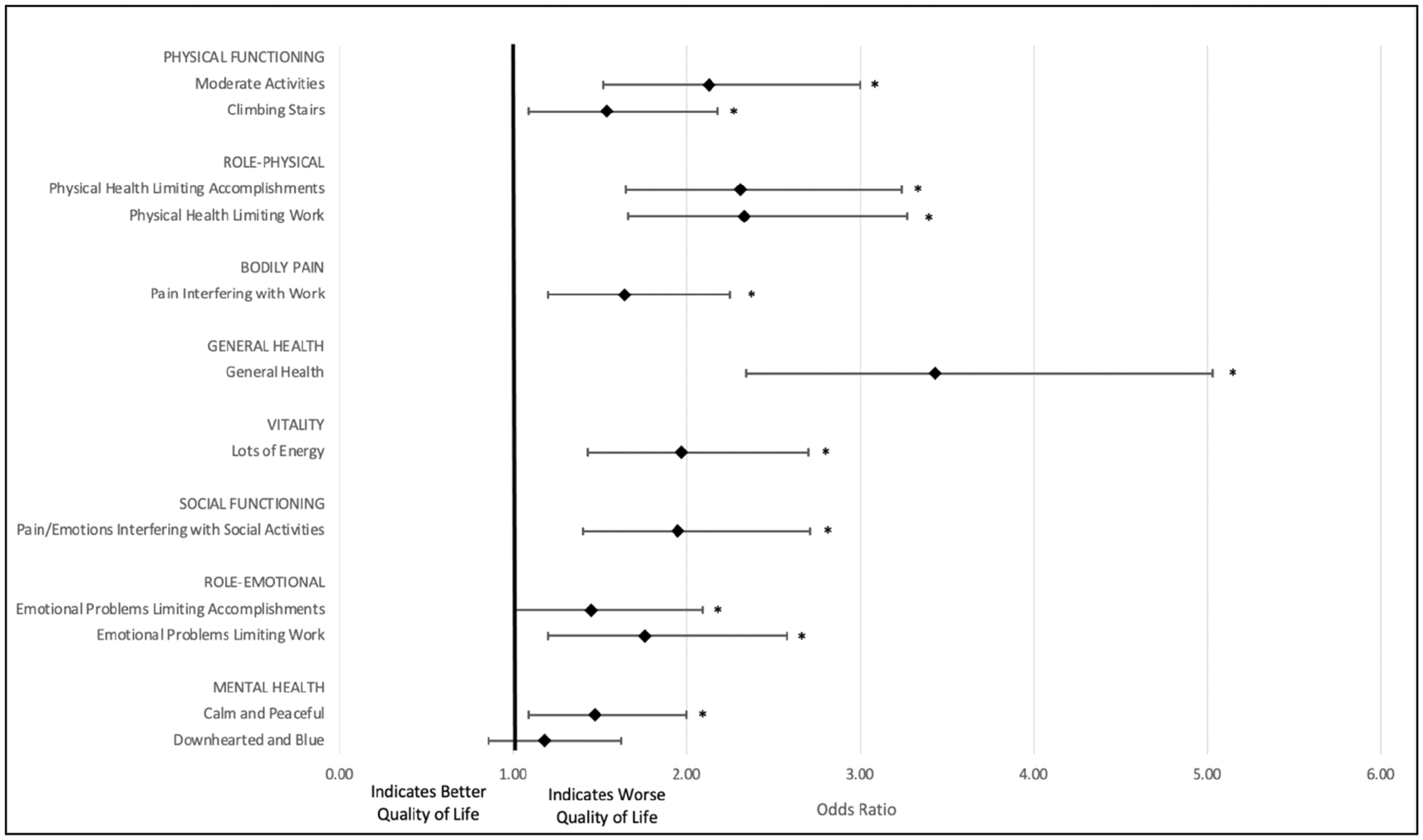
Odds of an inferior category response for cohort undergoing colorectal cancer treatment.

For those undergoing CRC treatment, the odds of an inferior response were statistically significant for all PCS dominant items, with the largest effect occurring within the general health (OR = 3.43, *p* < 0.001) and role-physical domains (OR = 2.33, *p* < 0.001 and OR = 2.31, *p* < 0.001). The odds of an inferior response were statistically significant for all MCS dominant items, except for questions specific to feeling “downhearted and blue”. The largest effect occurred within the vitality (OR = 1.97, *p* < 0.001) and social functioning (OR = 1.95, *p* < 0.001) domains (Figure 2).

**FIGURE 3 |:**
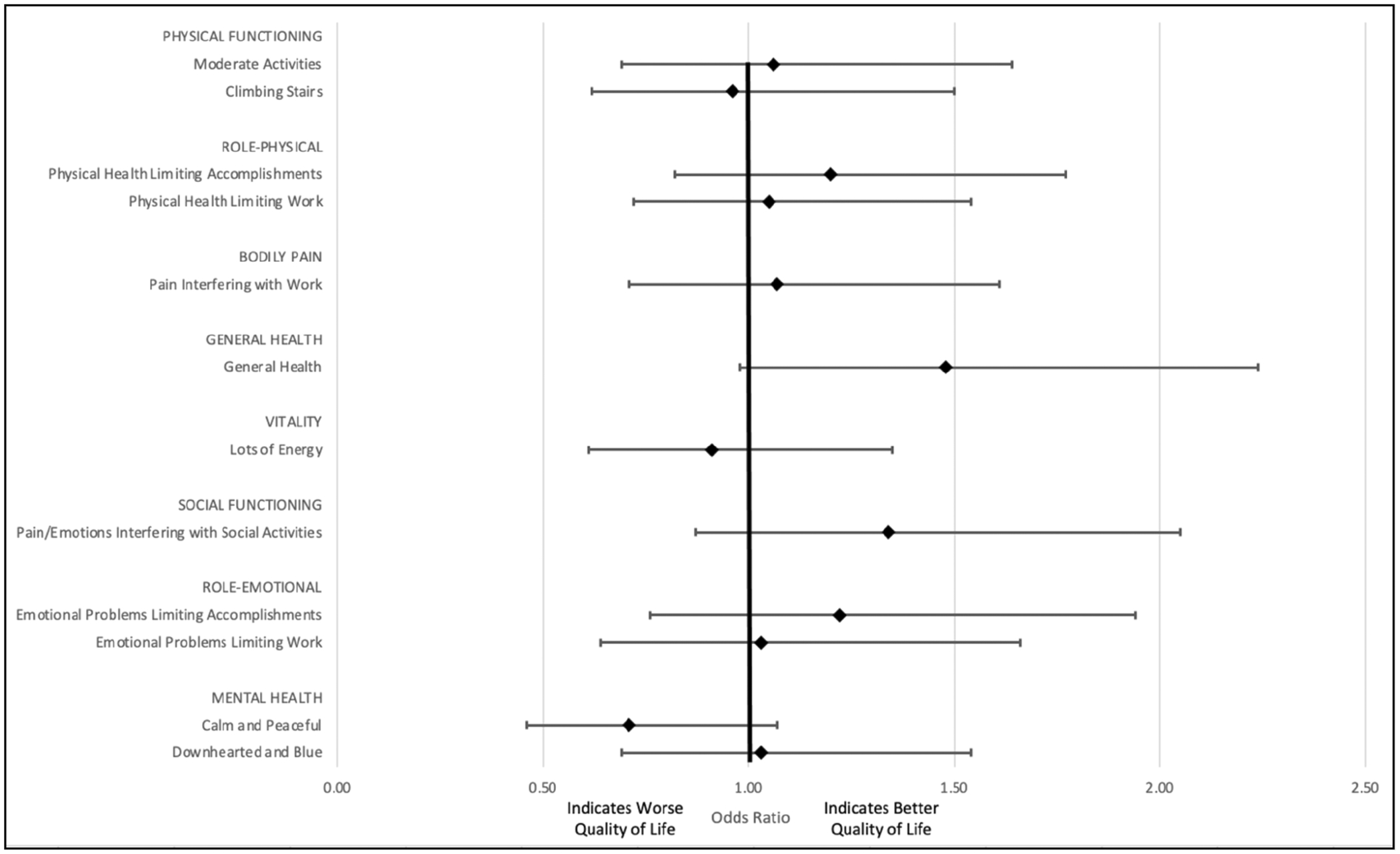
Odds of a superior category response for cohort who completed colorectal cancer treatment.

For those who completed CRC treatment, the odds of a superior category response at follow-up compared to baseline were calculated for each questionnaire item that contributes to PCS and MCS scores. This reflects the change in each item after completing CRC treatment. The odds of a superior response at follow-up were not statistically significant within any of the domains (Figure 3).

## 4 | Discussion

This longitudinal study examines HRQOL outcomes of MA beneficiaries who have received CRC treatment using the patient-reported MHOS survey. MA beneficiaries undergoing CRC treatment reported lower HRQOL with regard to both physical and mental health. Furthermore, up to 2 years after treatment completion, there was no change in HRQOL outcomes when compared to HRQOL at the time of treatment. This suggests that CRC treatment creates a new baseline quality of life status for older adults.

Our finding that CRC treatment has an overall negative impact on HRQOL is consistent with prior literature [18–20]. Factors associated with overall lower HRQOL in CRC survivors include being older, having comorbid conditions and unfavorable lifestyle behaviors, sociodemographic factors including social network size, income, and education, as well as experiencing a cancer recurrence [18, 20, 21]. Treatment modality may also influence HRQOL outcomes. With regard to surgical resection for CRC, a low rectal anastomosis and stoma creation have been associated with overall worse HRQOL [22–24], whereas chemotherapy and radiation therapy may have less of an impact on HRQOL outcomes in CRC patients [22, 25–27].

Furthermore, our adjusted analyses found that respondents who reported no CRC treatment at baseline scored 3.08 points lower on PCS scores and 1.40 points lower on MCS scores after initiating CRC treatment. The difference in PCS scores is clinically significant, whereas the difference in MCS scores only approaches the clinically significant standard of two points. This suggests that although CRC treatment affects HRQOL overall, it may have a more clinically significant impact on physical health rather than mental health. Previous literature has found evidence of the importance of physical activity for the health and well-being of cancer survivors [28–30], and specifically, the protective effect that physical activity has against poor physical HRQOL outcomes in CRC survivors [20]. Physical activity interventions such as community-based exercise programs have proven to be effective in improving physical health outcomes in cancer survivors [31, 32].

Before conducting this study, we had hypothesized that individuals receiving CRC treatment would show improvement in HRQOL outcomes once they were no longer were receiving CRC treatment; however, we found that this was not the case. Respondents who reported receiving CRC treatment at baseline showed no significant change in PCS or MCS scores after the completion of CRC treatment. This suggests that the effect CRC treatment has on physical and mental health carries on through the extended survivorship phase [33], with patients living at a new baseline health status. These findings are consistent with prior studies demonstrating the effects of CRC surgery on HRQOL outcomes [34, 35].

Given that there are evidence-based, effective interventions to restore HRQOL after CRC treatment, there might be opportunities to implement them broadly to mitigate the lack of recovery. For example, Gillis et al. found that initiating prehabilitation interventions in physical activity and nutrition counseling before surgical treatment of CRC was more effective in returning patients to baseline status when compared to initiating these interventions after surgery [36]. Additionally, several studies have found that patients partaking in at least 150 min of physical activity every week had sustained improvements on HRQOL over 2 years after their cancer diagnosis [29, 37].

In this study, individuals experienced significant degradation of their general health and limitations in physical performance, energy, and social functioning. Previous studies suggest that physical health limitations that impact the kind of work or other activities performed and limitations in social functioning are closely related to bowel function [38, 39]. Thus, our findings may be related to dysfunctional bowel symptoms associated with operative treatment of distal colorectal cancer, which includes fecal incontinence, frequency, and urgency [39]. On the other hand, limitations in energy have been associated with patients who underwent right-sided resection, which may be due to the anemia that is often present when diagnosed with right-sided colon cancer [35]. Therefore, it is important to consider the impact that tumor location-specific treatments can have on quality of life, as this could help to inform expectations during shared decision-making.

There are several limitations to this study. First, this is a retrospective cohort study of Medicare Advantage enrollees who responded to the survey, which may not fully represent the overall patient population and limits the generalizability of our findings. Second, the database lacks granularity with regard to treatment modality, which may be an important confounder for evaluating PCS and MCS scores. Third, due to the timing of survey completion, we are only able to follow each cohort over the span of 2 years, limiting our ability to capture changes in HRQOL over a longer period. Fourth, this study does not capture patients who died after initiating CRC treatment, which likely results in underestimating the effect that CRC treatment has on the change of baseline quality of life status. Lastly, the database does not provide cancer stage information, which limits our ability to adjust for this confounder, although future studies that link the MHOS to Medicare claims may be able to use predictive modeling methods to address this limitation [40].

In summary, CRC treatment can have an overall negative impact on HRQOL, but this influence may be more clinically significant with regard to physical health compared to mental health. Our findings emphasize the importance of informing patients of the long-term effects of CRC treatment with possible changes to baseline health status and support the implementation of prehabilitation interventions and physical activity programs to increase the chance of optimal and sustained recovery throughout the survivorship continuum.

## Supplementary Material

Supplement

Additional supporting information can be found online in the Supporting Information section.

## Figures and Tables

**TABLE 1 |:** Baseline demographics of MHOS respondents undergoing CRC treatment and those who have completed treatment.

	Cohort undergoing CRC treatment *n* = 368	Cohort who completed CRC treatment *n* = 206
Age, Mean (SD)	74.7 (6.8)	74.6 (6.9)
Sex, *n* (%)		
Male	177 (48.1)	> 95 (.)
Female	180 (48.9)	100 (48.5)
Did not respond	11 (3.0)	< 11 (.)
Race, *n* (%)		
Unknown or did not respond	19 (5.1)	< 11 (.)
White	289 (78.5)	140 (68.0)
Black	37 (10.1)	30 (14.6)
Other	11 (3.0)	< 11 (.)
Asian	12 (3.3)	< 11 (.)
American Indian or Alaska Native	0 (0.0)	< 11 (.)
Ethnicity, *n* (%)		
Hispanic	87 (23.6)	83 (40.3)
Not Hispanic	281 (76.4)	123 (59.7)
Primary language spoken, *n* (%)		
English	305 (82.9)	146 (70.9)
Spanish	35 (9.5)	38 (18.4)
Chinese	< 11 (.)	< 11 (.)
Some other language	< 11 (.)	< 11 (.)
Missing	14 (3.8)	> 11 (.)
Survey language, *n* (%)		
English	342 (92.9)	189 (91.7)
Spanish or Chinese	26 (7.1)	17 (8.3)
Proxy completed survey, *n* (%)		
Self	313 (85.1)	153 (74.3)
Proxy	33 (9.0)	30 (14.6)
Missing	22 (6.0)	23 (11.2)
Marital status, *n* (%)		
Married	186 (50.5)	88 (42.7)
Divorced or separated	65 (17.7)	49 (23.8)
Widowed	87 (23.6)	> 39 (.)
Never married	> 19 (.)	19 (9.2)
Did not respond	< 11 (.)	< 11 (.)
Education level, *n* (%)		
Did not graduate high school	> 86 (.)	70 (34.0)
High school graduate or GED	102 (27.7)	64 (31.1)
Some college or 2-year degree	90 (24.5)	39 (18.9)
4-year college degree or beyond	79 (21.5)	22 (10.7)
Did not respond	< 11 (.)	11 (5.3)
Household income, *n* (%)		
Income less than $10 000	47 (12.8)	46 (22.3)
Income $10 000–$19 999	73 (19.8)	46 (22.3)
Income $20 000–$29 999	46 (12.5)	25 (12.1)
Income $30 000–$49 999	55 (14.9)	28 (13.6)
Income $50 000 or more	68 (18.5)	19 (9.2)
Don’t know	42 (11.4)	29 (14.1)
Missing	37 (10.1)	13 (6.3)
Special needs, *n* (%)		
Chronic or disabling condition	< 11 (.)	< 11 (.)
Dual-eligible	67 (18.2)	50 (24.3)
Institutional	< 11 (.)	< 11 (.)
Not applicable/missing	> 281 (.)	> 134 (.)
Institutional status, *n* (%)		
Out of institution	> 357 (.)	> 195 (.)
Institutionalized	< 11 (.)	< 11 (.)
Hospice, *n* (%)		
No hospice start date present	368 (100.0)	206 (100.0)
Medicaid status, *n* (%)		
Out of Medicaid	260 (70.7)	127 (61.7)
In Medicaid (full or partial)	108 (29.3)	79 (38.3)
CMS region, *n* (%)		
Region 1 – Boston	21 (5.7)	< 11 (.)
Region 2 – New York	38 (10.3)	19 (9.2)
Region 3 – Philadelphia	37 (10.1)	20 (9.7)
Region 4 – Atlanta	51 (13.9)	27 (13.1)
Region 5 – Chicago	74 (20.1)	34 (16.5)
Region 6 – Dallas	31 (8.4)	23 (11.2)
Region 7 – Kansas City	> 21 (.)	< 11 (.)
Region 8 – Denver	< 11 (.)	< 11 (.)
Region 9 – San Francisco	45 (12.2)	38 (18.4)
Region 10 –Seattle	39 (10.6)	19 (9.2)

Abbreviations: CMS, Centers for Medicare and Medicaid Services; CRC, colorectal cancer; GED, general educational diploma; SD, standard deviation.

**TABLE 2 |:** Baseline health conditions of MHOS respondents undergoing CRC treatment and those who have completed treatment.

	Cohort undergoing CRC treatment *n* = 368	Cohort who completed CRC treatment *n* = 206
BMI category, *n* (%)		
Underweight (< 18.5)	14 (3.8)	< 11 (.)
Normal (18.5– < 25)	83 (22.6)	38 (18.4)
Overweight (25– < 30)	112 (30.4)	59 (28.6)
Obese (30 +)	130 (35.3)	77 (37.4)
Did not report	29 (7.9)	> 21 (.)
Current smoking status, *n* (%)		
Smoker	48 (13.0)	24 (11.7)
Nonsmoker	> 309 (.)	> 171 (.)
Did not report	< 11 (.)	< 11 (.)
Hypertension, *n* (%)		
Yes	246 (66.8)	152 (73.8)
No	> 111 (.)	54 (26.2)
Did not report	< 11 (.)	< 11 (.)
Angina pectoris or coronary artery disease, *n* (%)		
Yes	46 (12.5)	30 (14.6)
No	> 311 (.)	> 165 (.)
Did not report	< 11 (.)	< 11 (.)
Congestive heart failure, *n* (%)		
Yes	40 (10.9)	28 (13.6)
No	> 317 (.)	> 167 (.)
Did not report	< 11 (.)	< 11 (.)
Myocardial infarction or heart attack, *n* (%)		
Yes	29 (7.9)	23 (11.2)
No	> 328 (.)	> 172 (.)
Did not report	< 11 (.)	< 11 (.)
Other heart conditions, *n* (%)		
Yes	89 (24.2)	41 (19.9)
No	279 (75.8)	> 154 (.)
Did not report		< 11 (.)
Stroke, *n* (%)		
Yes	25 (6.8)	17 (8.3)
No	> 332 (.)	> 178 (.)
Did not report	< 11 (.)	< 11 (.)
Emphysema, asthma, or COPD, *n* (%)		
Yes	65 (17.7)	41 (19.9)
No	> 292 (.)	> 154 (.)
Did not report	< 11 (.)	< 11 (.)
Inflammatory bowel diseases, *n* (%)		
Yes	41 (11.1)	31 (15.0)
No	> 316 (.)	> 164 (.)
Did not report	< 11 (.)	< 11 (.)
ESRD, *n* (%)		
Yes	< 11 (.)	< 11 (.)
No	> 357 (.)	> 195 (.)
Arthritis of hip or knee, *n* (%)		
Yes	157 (42.7)	107 (51.9)
No	> 200 (.)	> 88 (.)
Did not report	< 11 (.)	< 11 (.)
Arthritis of hand or wrist, *n* (%)		
Yes	136 (37.0)	90 (43.7)
No	> 221 (.)	> 105 (.)
Did not report	< 11 (.)	< 11 (.)
Osteoporosis, or thin/brittle bones, *n* (%)		
Yes	72 (19.6)	49 (23.8)
No	> 285 (.)	> 146 (.)
Did not report	< 11 (.)	< 11 (.)
Sciatica, or pain/numbness traveling down leg, *n* (%)		
Yes	99 (26.9)	68 (33.0)
No	> 258 (.)	> 127 (.)
Did not report	< 11 (.)	< 11 (.)
Diabetes, or high blood sugar, or sugar in the urine, *n* (%)		
Yes	119 (32.3)	77 (37.4)
No	> 230 (.)	>118 (.)
Did not report	< 11 (.)	< 11 (.)

Abbreviations: BMI, body mass index; COPD, chronic obstructive pulmonary disease; ESRD, end stage renal disease.

**TABLE 3 |:** Unadjusted outcomes.

		Cohort undergoing CRC treatment	Cohort who completed CRC treatment
Physical component summary (PCS) Score, mean (SD)	Baseline	39.27 (11.80)	38.53 (11.90)
	Follow-up	36.18 (12.25)	36.74 (11.90)
Mental component summary (MCS) score, mean (SD)	Baseline	52.87 (10.67)	52.21 (10.96)
	Follow-up	51.47 (11.53)	51.34 (11.27)

Abbreviation: SD, standard deviation.

**TABLE 4 |:** Adjusted differences in baseline and follow-up survey outcomes.

	*β* coefficient [95% Confidence Interval]	*p* value
Cohort undergoing colorectal cancer treatment		
Physical Component Summary (PCS) score	−3.08 [−4.16, −2.01]	< 0.001
Mental Component Summary (MCS) score	−1.40 [−2.44, −0.36]	0.008
Cohort that completed colorectal cancer treatment		
Physical Component Summary (PCS) score	0.54 [−0.91, 1.98]	0.466
Mental Component Summary (MCS) score	0.07 [−1.44, 1.59]	0.924

*Note:* Model clustered by respondent identifier and adjusted for age, sex, race, ethnicity, primary language, survey language, proxy survey completion, marital status, education level, median household income, special needs, institutional status, Medicaid status, CMS region, BMI, smoking status, and baseline comorbidities.

## Data Availability

The data underlying this study are derived from the Medicare Health Outcomes Survey (MHOS), which is available through the Centers for Medicare & Medicaid Services (CMS). Access to the Medicare HOS data is restricted and requires approval from CMS to ensure compliance with legal and ethical standards, including the protection of patient confidentiality. Researchers may apply for access to the Medicare HOS data by submitting a data use agreement and following CMS’s procedures.

## Abbreviations:

CRC: colorectal cancer
HRQOL: health-related quality of life
MA: Medicare advantage
MCS: mental component score
MHOS: medicare health outcomes survey
PCS: physical component score
