# Supplementary material for: The Burden of Colorectal Cancer Treatment on Quality of Life: A Paired Longitudinal Analysis of Medicare Advantage Enrollees
Source: J Surg Oncol. Author manuscript; Available in PMC 2026 Jun 2. (PMC13007000; doi:10.1002/jso.28161)
Supplement: Supplement [file NIHMS2142157-supplement-Supplement.docx]

**Supplementary Table 1:** Medicare Health Outcomes Survey physical and mental component summary score dominant domains and their descriptions.

**Source**: National Committee for Quality Assurance. Healthcare Effectiveness Data and Information Set (HEDIS®) 2020 Volume 6: Specifications for the Medicare Health Outcomes Survey. Washington, DC. 2020. <https://hosonline.org/globalassets/hos-online/survey-administration/hos_hedis_volume6_2020.pdf>

| **Physical Component Summary (PCS) Dominant Domains** | |
| --- | --- |
| **Physical Functioning** (Survey Questions 2a, 2b) | Two questions ask respondents to indicate the extent to which their health limits their physical activities. |
| **Role-Physical**  (Survey Questions 3a, 3b) | Two questions ask respondents whether their physical health limits them in the kind of work or other usual activities they perform, in terms of time and performance. |
| **Bodily Pain**  (Survey Question 5) | One question asks respondents to indicate the extent to which pain interferes with the respondent’s normal activities. |
| **General Health**  (Survey Question 1) | One question asks respondents to rate their current, overall health status. |
| **Mental Component Summary (MCS) Dominant Domains** | |
| **Vitality**  (Survey Questions 6b) | One question asks respondents to rate their well-being by indicating how frequently they experience energy. |
| **Social Functioning**  (Survey Question 7) | One question asks respondents to indicate limitations in social functioning that result specifically because of their health. |
| **Role-Emotional**  (Survey Questions 4a, 4b) | Two questions ask respondents if emotional problems have caused them to accomplish less in their work or other usual activities, in terms of time and performance. |
| **Mental Health**  (Survey Questions 6a, 6c) | Two questions ask respondents how frequently they felt calm and peaceful, and how frequently they felt downhearted and blue. |
